# Antibiotic use in respiratory syncytial virus-positive children under two years: a prospective observational study at a tertiary hospital in Ghana

**DOI:** 10.3389/fpubh.2026.1696374

**Published:** 2026-03-20

**Authors:** Joycelyn Assimeng Dame, Kwabena Agyapong Osman, An Nguyen, Farina Shaaban, Evangeline Obodai, Clint Pecenka, Louis Bont, Bamenla Goka

**Affiliations:** 1 Department of Child Health, University of Ghana Medical School, College of Health Sciences, Accra, Ghana; 2 Center for Vaccine Innovation and Access, PATH, Ho Chi Minh City, Vietnam; 3 Department of Paediatrics, University Medical Centre Utrecht, Utrecht, Netherlands; 4 Virology Department, Noguchi Memorial Institute for Medical Research, College of Health Sciences, University of Ghana Legon, Accra, Ghana; 5 Center for Vaccine Innovation and Access, PATH, Seattle, WA, United States

**Keywords:** AMR (antimicrobial resistance), antibiotics, antimicrobial stewardship (AMS), children, Ghana, maternal RSV vaccine, RSV (respiratory syncytial virus)

## Abstract

**Introduction:**

Respiratory syncytial virus (RSV) is a major cause of lower respiratory tract infections in children, often leading to hospitalization in infants and vulnerable children. In low-resource settings where routine RSV diagnostics are unavailable, the overlap between clinical symptoms of RSV and those of bacterial pneumonia leads to unnecessary antibiotic use and contributes to the development of antimicrobial resistance. We conducted this study to evaluate the frequency and clinical determinants of antibiotic use among RSV-positive children under 2 years at a tertiary hospital in Ghana.

**Methods:**

We conducted a prospective observational sub-study nested within a longitudinal cohort of children at the Department of Child Health, Korle Bu Teaching Hospital, from June to November 2023. Eligible children aged <2 years with acute respiratory illness were enrolled and tested for RSV using molecular point-of-care testing. Antibiotic use and clinical characteristics were analyzed.

**Results:**

Of the 128 children enrolled, 72 (56.2%) tested positive for RSV. RSV-positive infants were significantly younger than RSV-negative infants (0–6 months vs. >6 months; *p* = 0.02). Among children with confirmed RSV infection, 48 (66.7%) received a total of 78 antibiotic prescriptions, although only 11 (23%) had clinically suspected bacterial co-infections. The most frequently prescribed antibiotic class was penicillins (38/78; 48.7%), followed by third-generation cephalosporins (17/78; 21.8%). Antibiotic use was significantly associated with indicators of disease severity, including hypoxia (*p* = 0.009), tachypnea (*p* = 0.015), dyspnea (*p* < 0.001), and hospital admission (*p* < 0.001). Independent predictors of antibiotic use were difficulty in breathing (OR 33.0, 95% CI 4.2–263.4; *p* = 0.001) and tachypnea (OR 9.3, 95% CI 1.4–60.9; *p* = 0.02).

**Discussion:**

Most RSV-positive children received antibiotics, often without confirmed bacterial co-infection. This highlights the need for antimicrobial stewardship, rapid diagnostics, and preventive strategies, such as maternal RSV vaccination, to reduce unnecessary antibiotic use and combat antimicrobial resistance in low-resource pediatric settings.

## 1 Introduction

Respiratory syncytial virus (RSV) is a leading cause of acute respiratory tract infections across all age groups. While it often results in mild symptoms, it can cause severe illness in young children, older adults, and immunocompromised individuals (1, 2). In children, RSV commonly affects the bronchioles, leading to airway obstruction and bronchiolitis (3). The clinical presentation includes fever, cough, tachypnea, increased work of breathing, wheezing, and crackles- symptoms that can overlap with those of other lower respiratory tract infections, such as pneumonia.

RSV bronchiolitis severity ranges from mild to severe. Children with mild disease are typically managed at home, while those with moderate to severe symptoms often require hospitalization for supportive care, including respiratory support (4). In high-resource settings, rapid diagnostic tests are readily available to confirm RSV infection, aiding appropriate clinical decision-making. However, in low-resource settings, such diagnostic tools are often unavailable. As a result, children with viral respiratory infections are frequently treated empirically with antibiotics, especially when hospitalized (5).

While antibiotics may be warranted in critically ill children with suspected bacterial co-infections (5), their routine use in viral infections, such as RSV bronchiolitis, is generally unnecessary and contributes to the growing global threat of antimicrobial resistance (AMR). Inappropriate antibiotic use increases the risk of resistance, which poses significant health and economic burdens. In 2019 alone, bacterial AMR was directly responsible for an estimated 1.27 million deaths and contributed to nearly 4.95 million deaths worldwide (6).

We aimed to evaluate antibiotic use and its clinical determinants in children with RSV-positive respiratory illness under 2 years of age presenting at the Department of Child Health (DCH), Korle Bu Teaching Hospital (KBTH), a tertiary facility in a low-resource setting where RSV testing is not routinely performed. Understanding prescribing patterns in the absence of routine RSV diagnostics is essential to inform targeted antimicrobial stewardship interventions and improve care.

## 2 Materials and methods

### 2.1 Study setting

We conducted a prospective observational sub-study nested within the Ghana cohort of the RSV GOLD III Health Economics Study- an international, prospective, multicenter study aimed at quantifying the direct medical, non-medical, and indirect costs associated with RSV infections in both hospitalized and non-hospitalized children under the age of two. The Ghanaian arm of the study was conducted at the DCH in KBTH, Accra, from June to November 2023, a period that typically coincides with the seasonal peak of RSV transmission in the region (7). We assessed antibiotic use during the study period by systematically abstracting prescribing information from the clinical notes of enrolled participants, particularly children with confirmed RSV infection.

KBTH serves as a major tertiary referral centre for southern Ghana and is the third-largest referral hospital on the African continent, with approximately 2,000 beds and 21 clinical and diagnostic departments. DCH provides comprehensive services, including outpatient consultations, emergency care, and inpatient management. Annually, it cares for an estimated 25,000 pediatric patients with a wide range of medical conditions. All pediatric admissions are routed through the department’s emergency unit for triage before onward admission.

Participants for the study were recruited from the outpatient clinic, emergency room, general pediatric wards, and the Pediatric Intensive Care Unit (PICU). Ethical approval was obtained from the KBTH Institutional Review Board (No. 000100/2023), and written informed consent was obtained from parents or legal guardians prior to enrollment.

### 2.2 Study population

Children eligible for inclusion in this study were under 2 years of age and met the World Health Organization (WHO) extended case definition for (severe) acute respiratory infection [(S)ARI] (8). Acute respiratory infection (ARI) was defined by the presence of respiratory symptoms- such as cough or shortness of breath- with sudden onset within the preceding 10 days. Requiring hospitalization defined the criteria for severe ARI; therefore, all hospitalized patients were categorized as severe, and participants exclusively seen as outpatients were categorized as non-severe. Infants younger than 4 days old were excluded to reduce the risk of misclassification and because facility-acquired infection cannot be ruled out, as were children whose clinical presentation was unrelated to respiratory illness or whose respiratory symptoms were attributed to non-infectious causes.

### 2.3 RSV diagnosis

Participating children were tested for RSV using a molecular point-of-care (POC) diagnostic device. A nasal swab was collected by a trained research assistant, and the sample was tested for RSV using the highly sensitive and specific POC device, ID NOW RSV (Abbott, Scarborough, ME, USA) (9). For outpatient participants, samples were obtained during the clinic visit; for admitted patients, sampling was performed once they were clinically stable, to avoid interfering with acute management. POC testing was an exclusively research practice, separate from immediate care, and therefore did not interfere with the patient’s diagnostic examination. To minimize the risk of detecting facility-acquired RSV infection, POC testing was performed as soon as possible after admission once the patient was stable. However, all samples were processed within 72 h of presentation. The 72-h testing window covered out-of-hours admissions, including weekend admissions.

Reverse transcriptase-polymerase chain reaction (RT-PCR) testing of all samples was conducted at the Noguchi Memorial Institute for Medical Research (NMIMR) to confirm the presence of RSV. For this, nasal samples were stored in a transport medium and frozen at −18 °C for later transport on an ice pack to NMIMR. Upon receipt in the laboratory, specimens were processed according to the Centers for Disease Control and Prevention (CDC) guidelines for respiratory syncytial virus (RSV) testing. Total RNA was extracted from respiratory samples using the QIAamp Viral RNA Mini Kit (QIAGEN, Germany) according to the manufacturer’s instructions. RSV detection was performed using real-time reverse transcription polymerase chain reaction (rRT-PCR), the CDC-recommended method due to its high sensitivity and specificity. The assay targeted conserved regions of the RSV nucleoprotein (N) gene using CDC-designed primers and probes. Each reaction included 5 μL of extracted RNA in a 20 μL reaction mixture containing RSV-specific primers, probe, reverse transcriptase buffer, enzyme mix, and nuclease-free water. Thermal cycling conditions consisted of reverse transcription at 50 °C for 30 min, initial denaturation at 95 °C for 5 min, followed by 45 cycles of denaturation at 95 °C for 15 s and annealing/extension at 60 °C for 30 s. Positive and negative controls were included in each run to ensure assay validity. Results were interpreted according to CDC criteria, and amplification curves confirmed RSV positivity. A test was considered RSV-positive either by POC testing and or RT-PCR. All test results were communicated to managing clinicians as they became available. Empirical antibiotics were initiated at the clinicians’ discretion and were not deferred pending RSV results, as RSV testing was conducted for research purposes and was not part of routine clinical care.

### 2.4 Data collection

Eligible participants were enrolled consecutively after their caregivers were informed of the study protocol and provided written informed consent. One eligible participant was not enrolled because the caregiver declined participation. For those who consented, a trained research assistant administered a structured questionnaire to the caregiver, collecting demographic information, including the child’s age, sex, and any known underlying medical conditions.

Clinical information was extracted from the hospital’s electronic medical records using a standardized data abstraction form. This included details on clinical presentation, antibiotic prescriptions, indications for antibiotic use and number of antibiotics prescribed. Anti-tuberculous drugs and topical medications were excluded.

Pneumonia was defined as a physician-diagnosed illness based on compatible respiratory symptoms and clinical signs, supported by radiological confirmation on chest X-ray.

Prescribed antibiotics were classified according to the WHO AWaRe categorization system (10), which groups antibiotics into Access, Watch, or Reserve categories based on their spectrum, indication, and potential for resistance.

### 2.5 Statistical analysis

Data were collected and entered into Excel and Castor electronic data capture system files (11), then merged to Stata (Stata Corporation LLC, College Station, TX, USA) (12) dataset for analysis using standard descriptive statistics. Comparisons were made between RSV-positive patients who received antibiotics and those who did not, using the Mann–Whitney test to compare medians and the χ^2^ test to compare categorical variables. Multivariable logistic regression was performed to predict antibiotic use among RSV-positive patients. Assumptions of logistic regression, including independence of data, multicollinearity, and nonlinearity of continuous independent variables, were checked using Pearson, Spearman, and Box-Tidwell tests. We used bidirectional stepwise regression to select variables for the model and the Hosmer-Lemeshow test to determine the goodness of fit. A *p*-value of less than 0.05 was considered statistically significant.

## 3 Results

### 3.1 Clinical characteristics of study participants with RSV infection

Between June and November 30, 2023, a total of 129 eligible children were identified, with 128 enrolled after obtaining informed consent Table 1. Of these, 72 (56.2%) tested positive for RSV. Fourteen cases initially reported as negative by POC testing were later confirmed positive by RT-PCR at NMIMR and included as RSV-positive. Two children died, one with RSV and one without. Children with RSV were younger (0–6 months vs. > 6 months, *p* = 0.02) and more likely to present with tachypnea (*p* = 0.046). Among those who tested positive for RSV, 48 (66.7%) received antibiotics during their illness Table 1.

**Table 1:** Clinical characteristics of children categorized by their RSV diagnosis.

Variables	RSV-positive	RSV-negative	Total	*p*-value^a^
	*N* = 72 (56.2%)	*N* = 56 (43.8%)	*N* = 128 (100%)	
Median age in months (IQR)	2.9 (1.6–5.8)	5.1 (1.6–12.6)	3.4 (1.6–8.1)	0.045
Age categories				0.020
0–6 months	55 (64.7%)	30 (35.3%)	85 (100%)	
>6–12 months	9 (45.0%)	11 (55.0%)	20 (100%)	
>12 months	8 (34.8%)	15 (65.2%)	23 (100%)	
Sex				0.249
Male	39 (52.0%)	36 (48.0%)	75(100%)	
Female	33 (62.3%)	20 (37.7%)	53(100%)	
Prematurity				0.411
Born preterm^b^	20 (62.5%)	12 (37.5%)	32 (100%)	
Born term^c^	52 (54.2%)	44 (45.8%)	96 (100%)	
Diagnosis				0.173
Bronchiolitis	5 (45.5%)	6 (55.5%)	11 (100%)	
Pneumonia	35 (64.8%)	19 (35.2%)	54 (100%)	
URTI^d^	29 (54.7%)	24 (45.3%)	53 (100%)	
Other^e^	3 (30.0%)	7 (70.0%)	10 (100%)	
Comorbidity				0.422
None	56 (60.2%)	37 (39.8%)	93 (100%)	
Congenital heart disease	1 (20.0%)	4 (80.0%)	5 (100%)	
Neurodevelopmental disease	1 (50.0%)	1 (50.0%)	2 (100%)	
Infection diagnosis^f^	6 (46.1%)	7 (53.9%)	13 (100%)	
Non-infection diagnosis^g^	8 (53.3%)	7 (46.7%)	15 (100%)	
Temperature/°C				0.481
36– < 37.5	54 (54.0%)	46 (46.0%)	100 (100%)	
37.5–38.5	12 (60.0%)	8 (40.0%)	20 (100%)	
>38.5	6 (75.0%)	2 (25.0%)	8 (100%)	
Oxygen saturation/%				0.747
95–100	57 (54.8%)	47 (45.2%)	104 (100%)	
90–94	4 (57.1%)	3 (42.9%)	7 (100%)	
<90	11 (64.7%)	6 (35.3%)	17 (100%)	
Tachypnea^h^ on arrival				0.046
Tachypnea	32(69.6%)	14 (30.4%)	46 (100%)	
No tachypnea	37 (50.7%)	36 (49.3%)	73 (100%)	
No record	3 (33.3%)	6 (66.7%)	9 (100%)	
Difficulty in breathing on arrival				0.249
Difficulty in breathing	33 (62.3%)	20 (37.7%)	53 (100%)	
No difficulty in breathing	39 (52.0%)	36 (48.0%)	75 (100%)	
Antibiotics use				0.486
Yes	48 (58.5)	34 (41.5)	82 (100)	
No	24 (52.2)	22 (47.8)	46 (100)	
Admission for this illness				0.434
Inpatient	41 (59.4%)	28 (40.6)	69 (100%)	
Outpatient	31 (52.5%)	28 (47.5%)	59 (100%)	

^a^*p*-value–chi-squared analysis or Fisher’s test for categorical variables, and the Mann–Whitney test for the median age. Statistical significance was set at *p* ≤ 0.05.

^b^Born preterm–born before 37 completed weeks of gestation.

^c^Born term–born anytime after 37 completed weeks of gestation, to include post-term.

^d^URTI-upper respiratory tract infection.

^e^Other primary diagnoses include aspiration pneumonia and other non-respiratory diagnoses, including acute gastroenteritis, adenoid hypertrophy, acute conjunctivitis, and inguinal hernia.

^f^Infection diagnoses include acute gastroenteritis, sepsis, and urinary tract infection.

^g^Non-infection diagnoses include rectovaginal fistula, subgaleal hematoma, infantile haemangioma, G6PD deficiency, anaemia, Hirschsprung disease, and laryngomalacia.

^h^Tachpnea–defined by age [4 weeks–2 months = > 60 cpm; >2 months −1 year = > 50 cpm; > 1 year = > 40 cpm].

### 3.2 Clinical factors associated with antibiotic use among RSV-positive children

Among the 72 children with confirmed RSV infection, 48 (66.7%) received antibiotics during their illness. Children who received antibiotics were more likely to present with lower oxygen saturation (*p* = 0.009), tachypnea (*p* = 0.015), difficulty in breathing (*p* < 0.001), or require hospitalization (*p* < 0.001) than those who did not receive antibiotics. Those who received antibiotics during their hospitalization were also more likely to be prescribed antibiotics after discharge (*p* = 0.021). Prior antibiotic use before hospital arrival did not differ significantly between the two groups Table 2. Independent predictors of antibiotic use were difficulty in breathing (OR 33.0, 95% CI 4.2–263.4; *p*  = 0.001) and tachypnea (OR 9.3, 95% CI 1.4–60.9; (*p*  = 0.02) (Table 3).

**Table 2:** Clinical factors associated with antibiotic use among RSV-positive children.

Variable	Use of antibiotics in this illness	No use of antibiotics in this illness	Total = 72 (100%)	*p*-value^a^
	*N* = 48 (66.7%)	*N*/ = 24 (33.3%)		
Median age/months (IQR)	2.6 (1.5, 6.0)	3.4 (1.6, 5.4)	2.9 (1.6–5.8)	0.716
Age by categories/months (%)				0.867
0–6	36 (65.5)	19 (0.9)	55 (100)	
>6–12	6 (66.7)	3 (33.3)	9 (100)	
>12	6 (75.0)	2 (25.0)	8 (100)	
Sex				0.616
Male	25 (64.1)	14 (35.9)	39 (100)	
Female	23 (69.7)	10 (30.3)	33 (100)	
Prematurity				0.852
Born preterm	13 (65.0)	7 (35.0)	20 (100)	
Born term	35 (67.3)	17 (32.7)	52 (100)	
Temperature at presentation/°C				0.127
36–37.5	33 (61.1)	21 (38.9)	54 (100)	
>37.5–38.5	11 (91.7)	1 (8.3)	12 (100)	
>38.5	4 (66.7)	2 (33.3)	6 (100)	
Oxygen saturation at presentation				0.009
95–100%	33 (57.9)	24 (42.1)	57 (100)	
94–90%	4 (100)	0 (0)	4 (100)	
<90%	11 (100)	0 (0)	11 (100)	
Tachypnea on arrival				0.015
Tachypnea	27 (84.4)	5 (15.6)	32 (100)	
No tachypnea	19 (51.4)	18 (48.6)	37 (100)	
Difficulty in breathing				<0.001
Difficulty in breathing	31 (93.9)	2 (6.1)	33 (100)	
No difficulty in breathing	17 (43.6)	22 (56.4)	39 (100)	
Admission for this illness				<0.001
Inpatient	41 (100)	0 (0)	41(100)	
Outpatient	7 (22.6)	24 (77.4)	31 (100)	
Antibiotic use before the current visit/hospitalization				0.177
Yes	10 (90.9)	1 (9.1)	11 (100)	
No	36 (62.1)	22 (37.9)	58 (100)	
Antibiotic use after visit/discharge				0.021
Yes	13 (92.9)	1 (7.1)	14 (100)	
No	35 (60.3)	23 (39.7)	58 (100)	
Co-infection diagnosed or suspected^b^				0.129
Yes	11 (84.6)	2 (15.4)	13 (100)	
No	37 (62.7)	22 (37.3)	59 (100)	

^a^Chi-squared analysis or Fisher’s test for categorical variables, and the Mann–Whitney test for the median age. Statistical significance was set at *p* ≤ 0.05.

^b^Include acute gastroenteritis, sepsis, suspected meningitis, otitis media, conjunctivitis, urinary tract infection, and aspiration pneumonia.

**Table 3:** Logistic regression model with antibiotic use as outcome variable.

Logistic regression model with antibiotic use as outcome variable		
Explanatory variables	Odds ratio (95% CI)	*p*-value
Difficulty in breathing	33.4 (4.2–263.4)	0.001
Tachypnea	9.3 (1.4–60.9)	0.020
Use of antibiotics before the visit	8.4 (0.6–110.0)	0.106
Co-infection diagnosed or suspected	6.7 (0.7–62.3)	0.097
Temperature at presentation/°C	0.1 (0.02–1.2)	0.078
Born preterm	0.3 (0.05–1.7)	0.168
Performance of the prediction model		
Accuracy (correctly classified)	92.42%	
Sensitivity	95.45%	
Specificity	86.36%	

### 3.3 Antibiotic use among 48 children with RSV infection

**Figure 1:**
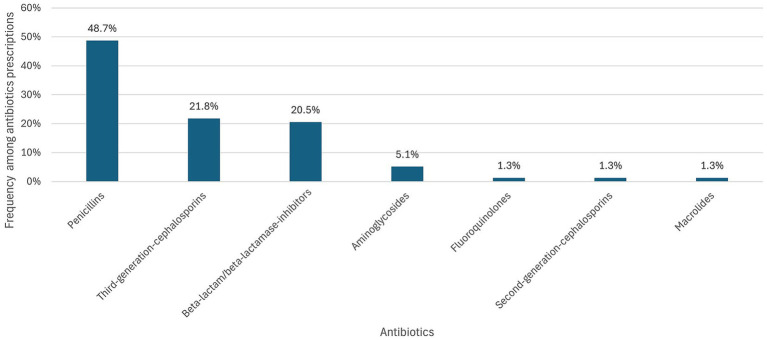
Frequency of antibiotics prescribed among RSV-positive children.

Among the 48 RSV-positive children who received antibiotics, 78 antibiotic prescriptions were recorded. The most frequently prescribed class was penicillins (48.7%), followed by third-generation cephalosporins (21.8%) Figure 1.

### 3.4 Antibiotics classified by the WHO 2023 AWaRE classification

**Figure 2:**
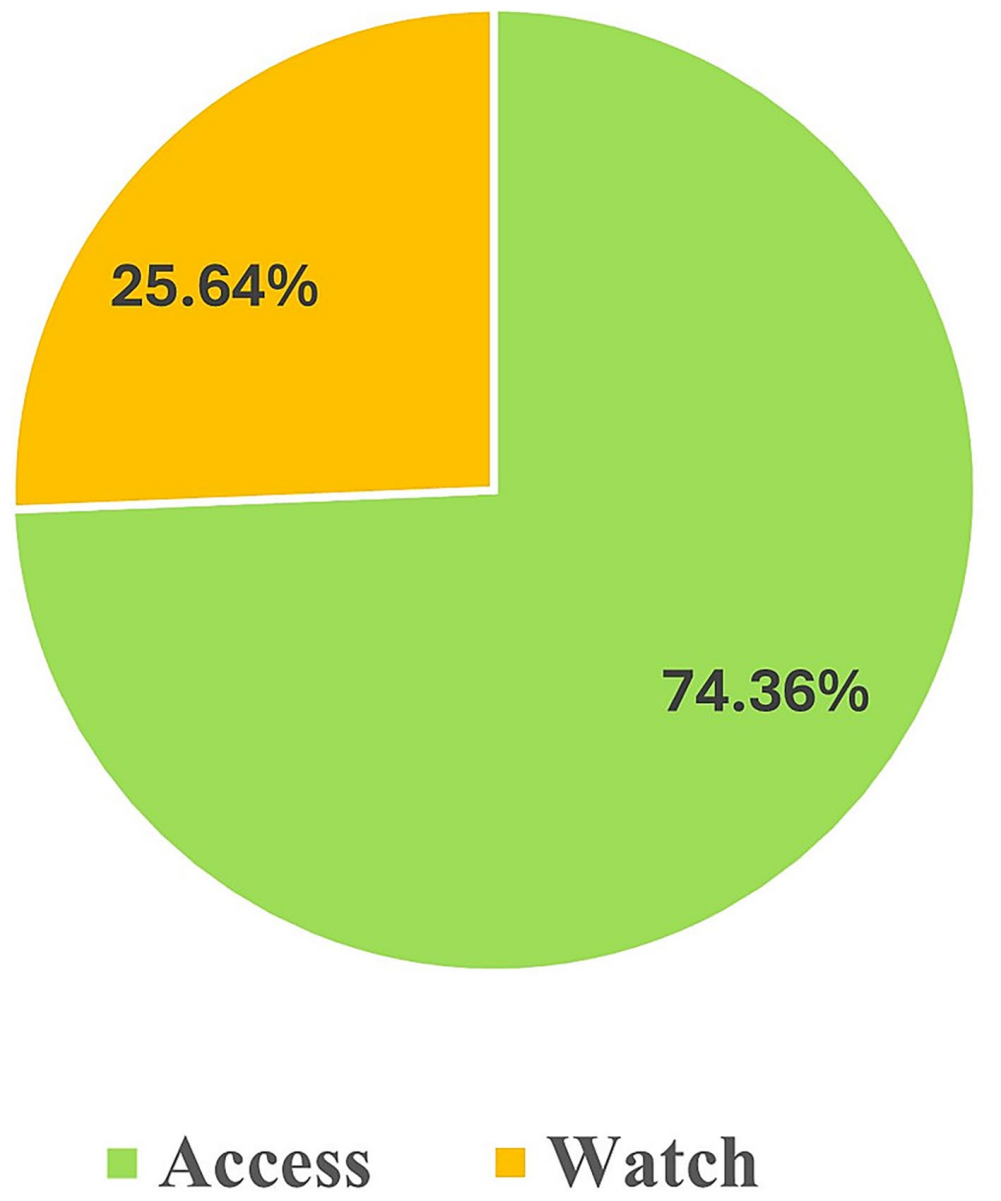
Seventy-eight antibiotic prescriptions classified according to the 2023 WHO AWaRe classification.

The majority of antibiotics used in children with RSV-positive infection were from the Access group (74.4%) Figure 2.

### 3.5 Co-infection status among RSV-positive children receiving antibiotics (*n* = 48)

Among the 48 RSV-positive children who received antibiotics, 11 (23%) had a documented non-respiratory co-infection, including acute gastroenteritis, urinary tract infection, or sepsis. In this group, the primary respiratory diagnoses were pneumonia (*n* = 7) and upper respiratory tract infection (URTI) (*n* = 4). The remaining 37 children (77%) had no identified co-infection. Their respiratory diagnoses included pneumonia (*n* = 28), URTI (*n* = 5), and bronchiolitis (*n* = 4).

## 4 Discussion

In our study, nearly 70% of children under 2 years of age with laboratory-confirmed RSV infection received antibiotics during their illness. This high rate of antibiotic use reflects a common challenge in low-resource settings, where clinical overlap between viral and bacterial respiratory infections, the absence of routine diagnostic tools, and a fear of missing bacterial co-infections often drive empirical antibiotic prescribing. The overuse of antibiotics has important implications for AMR. RSV is a viral illness, and unnecessary antibiotic exposure contributes to resistance by promoting selection pressure on gut and respiratory flora (13). This is particularly concerning in LMICs, where access to second-line antibiotics is limited, and treatment options for resistant infections are few. A systematic review by Luchen et al. of the impact of antibiotics on the gut microbiome of infants in LMICs showed that antibiotics significantly reduce the diversity and alter the composition of the infant gut microbiome, while simultaneously selecting for resistance genes that can persist for months after treatment (14).

Our findings underscore the urgent need to implement antimicrobial stewardship programs (ASPs) in pediatric departments, along with clear clinical pathways for managing viral illnesses, such as RSV. These programs should incorporate diagnostic testing and reinforce clinical guidelines that promote appropriate antibiotic use in accordance with the WHO AWaRe classification. They should also provide training for healthcare providers on rational prescribing and include ongoing monitoring of antibiotic utilization. Investment in such systems is critical to safeguard antibiotic efficacy and improve outcomes in vulnerable pediatric populations.

Antibiotic use was significantly associated with markers of disease severity, including tachypnea, low oxygen saturation, difficulty breathing, and inpatient admission, all of which can mimic bacterial pneumonia. Furthermore, the logistic regression model identified difficulty in breathing and tachypnea as independent predictors of antibiotic prescribing. These findings suggest that visible markers of respiratory distress strongly influence clinical decision-making, even in the absence of bacterial evidence. While this justifies the initiation of antibiotics in some cases, the absence of a documented bacterial co-infection during hospitalization, combined with our point-of-care molecular testing, would provide the diagnostic confidence to consider early discontinuation of antibiotics. While limited access to rapid viral testing may contribute to antibiotic use in some low-resource settings, diagnostic availability alone is unlikely to change prescribing behavior. A multifaceted antimicrobial stewardship approach, including education, guideline reinforcement, audit and feedback, and improved access to viral diagnostics, is therefore essential to optimize care for children with RSV infection. Our findings align with previous research. A prospective study conducted in Israel reported that 33.4% of children with confirmed RSV infection but no evidence of bacterial co-infection received unnecessary antibiotic treatment. The likelihood of inappropriate antibiotic use was higher in cases with bacterial cultures and in children presenting with signs of severe illness, including low oxygen saturation, elevated temperature, rapid breathing, and recent emergency department visits (15).

The use of Watch group antibiotics, particularly third-generation cephalosporins, among RSV-positive children is concerning, given that RSV is a viral illness and does not warrant routine antibiotic therapy, especially not with broad-spectrum agents that carry a higher risk of resistance (13). Thus, ASPs that incorporate AWaRe as a core framework for monitoring and optimizing pediatric antibiotic use are needed. Implementing hospital-based audit-feedback systems based on AWaRe could reduce the use of Watch group antibiotics and support the WHO’s target that at least 60% of national antibiotic consumption should come from the Access group (16). In our study, 74% of prescribed antibiotics belonged to the Access group, a figure that may seem less concerning, given that it exceeded the WHO’s 60% target. However, most respiratory infections were viral and did not require antibiotic treatment, highlighting inappropriate antibiotic use despite alignment with WHO’s prescribing benchmarks.

The availability of a molecular POC test within 72 h of presentation enabled the accurate identification of viral etiology and could have informed the duration of antibiotic use. POC testing is increasingly recognized as an essential component of diagnostic stewardship, enabling clinicians to distinguish between viral and bacterial infections at the bedside and reduce the use of empirical antibiotics (17). A randomized controlled trial in Vietnam demonstrated that C-reactive protein-guided antibiotic prescribing, when combined with viral testing, reduced antibiotic use by more than 40% among children with respiratory illnesses (18). Implementing similar stewardship strategies, including syndromic POC testing, could improve prescribing practices at KBTH and other tertiary centres in Ghana.

Importantly, these findings also strengthen the case for RSV prevention through maternal immunization and the use of monoclonal antibodies in infants. The WHO recommends that all countries introduce RSV immunization products to prevent severe disease in infants, using either maternal vaccination (RSVPreF) or infant monoclonal antibody (nirsevimab), depending on local context. Countries are to consider factors such as cost, supply, health system readiness, and implementation feasibility when choosing between the two products (19). By preventing severe illness, RSV immunization can reduce unnecessary antibiotic use, supporting its inclusion in national immunization programs as a strategy to combat AMR. Our findings reinforce this approach, particularly in settings with a high RSV burden and frequent empirical antibiotic prescribing.

### 4.1 Limitations

Although RSV was confirmed by molecular POC testing and RT-PCR, routine bacterial investigations and testing for other viral pathogens were not performed, potentially leading to underestimation of appropriate antibiotic use. Furthermore, the rationale for antibiotic prescribing was not explored, limiting our understanding of clinicians’ decision-making processes. The relatively small sample size of RSV-positive children may have affected the stability of the logistic regression model, leading to statistically significant predictors with wide confidence intervals (e.g., difficulty in breathing and tachypnea) and non-significant associations for other variables (e.g., suspected co-infection and fever; see Table 3).

As a single-center study conducted in a large urban tertiary hospital, the findings may not be fully generalizable to smaller or rural healthcare settings. Lastly, long-term outcomes, such as length of hospital stay and treatment response, were not assessed, limiting conclusions about the broader clinical impact of antibiotic prescribing practices.

## 5 Conclusion

Our study demonstrated a high prevalence of antibiotic use in RSV-positive children under 2 years, primarily driven by clinical severity. These findings highlight the need for ASPs, including diagnostic stewardship and preventive measures such as maternal RSV vaccination, to be integrated into pediatric care in Ghana and comparable low-resource settings. Tackling these challenges will require coordinated efforts across clinical practice, diagnostic capacity, and health policy.

## Data Availability

The raw data supporting the conclusions of this article will be made available by the authors, without undue reservation.
